# Early and Midterm Outcomes of Valve-Sparing Aortic Root Replacement—Reimplantation Technique

**DOI:** 10.1055/s-0039-1683383

**Published:** 2019-04-01

**Authors:** Marisa Cevasco, Siobhan McGurk, Maroun Yammine, Lokesh Sharma, Julius Ejiofor, Anthony Norman, Michael N. Singh, Prem Shekar

**Affiliations:** 1 Division of Cardiac Surgery, Brigham and Women's Hospital, Boston, Massachusetts; 2 Division of Cardiology, Brigham and Women's Hospital, Boston, Massachusetts

**Keywords:** valve-sparing aortic root replacement, David procedure, reimplantation technique

## Abstract

**Background** Valve-sparing aortic root replacement (VSARR) is an increasingly popular alternative to traditional aortic root replacement for aortic root aneurysm disease with a normal aortic valve. We evaluated the early and midterm outcomes of VSARR—reimplantation technique (VSARR-RT) done at a single institution over a decade.

**Materials and Methods** We performed a retrospective study of all patients who underwent VSARR-RT between January 2004 and July 2014.

**Results** A total of 85 patients underwent VSARR-RT. Median time to latest echocardiographic follow-up was 4 years (range: 15–72 months). Total observation time was 491 patient years. Mean age was 44.6 ± 14.3 years, and 13 (15%) were women. Thirty-nine (46%) patients had a connective tissue disorder and 6 (7%) had a bicuspid aortic valve. Thirty-three (39%) patients underwent concomitant procedures, including coronary artery bypass grafting ( *n* = 9, 11%), mitral valve repair ( *n* = 8, 9%), and aortic hemi-arch replacement ( *n* = 7, 8%). There were no operative deaths or in-house mortality and no postoperative strokes. Kaplan-Meier analysis demonstrated survival of 99% (95% confidence interval [CI]: 97–100%) at 2 years and 98% (95% CI: 97–100%) at 8 years. Freedom from reoperation was 95.8% (95% CI: 91.2–100%) at 8 years. Freedom from endocarditis was 100% at 8 years. At the last echocardiographic follow-up, 95% of patients were free of severe aortic regurgitation (AR) and 82% free of moderate AR. Of the four patients who had severe AR, three underwent reoperations and received prosthetic valves and one is being clinically monitored.

**Conclusion** This study reports early and midterm outcomes after VSARR-RT at our institution, including those patients who underwent a VSARR-RT procedure combined with other procedures. Further follow-up remains necessary to determine long-term outcomes.

## Introduction

Traditionally, aortic root aneurysm repair involved replacing the root with a composite valve graft conduit. The use of a mechanical valve graft conduit as described in the original Bentall-DeBono procedure is reliable and safe, with excellent long-term survival and a low rate of aortic reoperation. 1 However, this approach mandates anticoagulation for mechanical valves to prevent thromboembolic complications. Also, for patients receiving bioprosthetic valve graft conduits, it is associated with a certain rate of reoperation due to degeneration of bioprosthetic valves. 2

Valve-sparing aortic root replacement—reimplantation technique (VSARR-RT) (also known as the *David procedure* ) has become an increasingly popular alternative to composite valve graft replacement. David and Feindel originally described valve-sparing aortic root replacement (VSARR) in a 1992 case series. 3 Since their seminal paper, several studies have shown that the reimplantation technique of VSARR provides stable aortic valve function and is ideal for young patients with an aortic root aneurysm. 3 4 5 6 Early outcomes are good, with mortality rates as low as 0% and few operative complications. However, it is well known that the durability of this repair varies considerably. 3 6

Our institution first began performing VSARR-RT 12 years ago. In this article, we describe our experience with the VSARR-RT and analyze our midterm outcomes.

## Materials and Methods

With approval from our institutional review board, including waived informed consent, we retrospectively reviewed all patients undergoing VSARR-RT from January 2004 to July 2014; a total of 85 patients were identified. Patient characteristics, perioperative data, laboratory test results, and in-hospital outcomes were recorded at the time of the hospitalization and extracted from electronic medical records (EMRs). Variables were coded according to the Society for Thoracic Surgeons Adult Cardiac Surgery database specifications, version 2.52. Follow-up data were obtained from EMR or from an internal research data repository. Long-term survival data were obtained by institutional follow-up protocols and from our state Department of Public Health. Patients are seen for their initial postoperative follow-up by their surgeon and subsequently followed by their cardiologists and the department's clinical nurse practitioners. Our research team then accesses clinical information through the EMR or by directly contacting the patient's outpatient cardiologist. There were 98.8% (84/85) follow-up for survival and 94.1% follow-up for postoperative echocardiographic data. Survival time was calculated in months between the date of surgery and September 30, 2015 based on whether the patient was alive. There was a known date of death or date of last known clinical contact. Time to reintervention or evidence of aortic insufficiency (AI) was calculated in months from the date of surgery to the date of the event, date of the first abnormal echocardiographic report, or September 30, 2015.

Preoperative AI, as assessed by transthoracic or transesophageal echocardiography, was graded as none (0), trace/trivial (1), mild (2), moderate (3), or severe (4). Repair failure was defined as the development of any pseudoaneurysm or true aneurysm (i.e., coronary button site) involving the root, reoperation for graft infection or endocarditis, or moderate or greater aortic valve dysfunction. Reintervention included subsequent aortic valve surgery.

### Surgical Technique

After establishing cardiopulmonary bypass and arresting the heart, the aorta was transected perpendicular to its long axis, and the aortic sinuses were excised. A Valsalva graft (Gelweave Valsalva graft, Vascutek Ltd., Renfrewshire, Scotland) or Hemashield graft (Hemashield Gold Vascular graft, Maquet Getinge Group, Rastatt, Germany) of appropriate size was sutured to the aortic annulus and then the aortic valve was resuspended to the conduit. (Eight surgeons used a Valsalva graft; the ninth surgeon's preference was to use a straight Hemashield graft.) Nine patients underwent a leaflet plasty. This was performed by plication of the leaflet with a CV-5 Gore-Tex suture in eight of the patients, and in one patient, by weaving a 5–0 polypropylene suture along the free edge of the cusp and anchoring it to the commissure. Coronary ostial anastomoses were performed in the corresponding sinus, and distal aortic anastomosis was completed. The patient was weaned off cardiopulmonary bypass and closed in the standard fashion.

### Statistical Methods

Categorical variables are presented as percent and number ( *n* ) and were evaluated by Fisher's exact test. Continuous variables are presented as mean +/− standard deviation (SD, if normally distributed, or median and interquartile range (IQR), if non-normally distributed. Analyses of continuous normally distributed variables were done using Student's *t* -test with Levine's homogeneity of variance or Mann-Whitney's U test as appropriate. Survival and time to reintervention were estimated using Kaplan-Meier analyses. All analyses were conducted using IBM SPSS Statistics version 22.0 (IBM Corporation, Armonk, NY) and *p* ≤ 0.05 was the criterion for significance.

## Results

### Patient Characteristics

A total of 85 patients underwent VSARR-RT. Most patients were men (85%), with a mean age of 44.6 ± 14.3 years ( Table 1 ). No patients had previous cardiac surgery and 39 (46%) had connective tissue disorders; 24 had Marfan's disease, 6 had Loeys-Dietz, and 9 had a familial connective tissue disorder or known genetic component to their presentation.

**Table 1:** Demographics and characteristics of 85 patients undergoing VSARR-RT

Variables	Patients ( *n* = 85), No. (%)
Women	13 (15.3)
Hypertension	32 (37.6)
Diabetes	1 (1.2)
Renal failure	0 (0)
Peripheral vascular disease	10 (11.8)
Cerebrovascular disease	4 (4.7)
NYHA class III/IV	6 (7.1)
Connective tissue disorders	46 (54.1)
Indication for aortic surgery	
Aneurysm	81 (95.3)
Dissection	2 (2.4)
Bicuspid aortic valve	6 (7.1)
Aortic root diameter (mean, SD)	4.93 ± 0.68
Preoperative aortic regurgitation	
None	19 (22.4)
Trace	17 (20.0)
Mild	24 (28.2)
Moderate	17 (20.0)
Severe	8 (9.4)

Abbreviations: NYHA, New York Heart Association; SD, standard deviation; VSARR-RT, valve-sparing aortic root replacement—reimplantation technique.

### Etiology and Echocardiographic Findings

Four (4.8%) patients had acute dissections involving the ascending aorta whereas the remaining 81 (95%) had aneurysms. Mean aortic root diameter was 4.92 ± 0.7 cm. Six (7%) patients had a bicuspid aortic valve. Preoperatively, there was no AI in 19 (22%) patients, whereas 17 had moderate (20%) and 8 (9%) had severe AI ( Table 1 ).

### Operative and In-hospital Outcomes

Table 2 shows operative characteristics and in-hospital outcomes. Concomitant procedures included coronary artery bypass grafting ( *n* = 9, 11%), mitral valve repair ( *n* = 8, 9%), and hemi-arch replacement ( *n* = 7, 8%). Ascending aorta replacement was included in 57 patients whose aneurysm extended beyond the sinotubular junction but proximal to the takeoff of the innominate artery (67%). Median cardiopulmonary bypass time was 238 minutes (IQR = 213, 285) with a median aortic cross-clamp time of 196 minutes (IQR = 177, 239).

**Table 2:** Operative and In-hospital outcomes for 85 patients undergoing VSARR-RT

Variables	Values
Emergent operative status ( *n* , %)	4 (4.7)
Aorta procedures ( *n* , %)	
Ascending aorta replaced	57 (67.1)
Hemi-arch	7 (8.2)
Root graft size (mm) (median, IQR)	28, 30 (30)
Bypass time (min)	213, 285 (238)
Cross-clamp time (min)	177, 239 (196)
Postoperative IABP used ( *n* , %)	0 (0)
Reoperation for bleeding ( *n* , %)	1 (1.2)
Permanent stroke ( *n* , %)	0 (0)
New-onset renal failure ( *n* , %)	0 (0)
Ventilation time (h) (median, IQR)	3, 10 (6)
Ventilation > 24 h ( *n* , %)	6 (7.1)
ICU stay (h) (median, IQR)	22, 48 (26)
LOS (days) (median, IQR)	5, 7 (6)
Operative mortality ( *n* , %)	0

Abbreviations: IABP, intra-aortic balloon pump; ICU, intensive care unit; IQR, interquartile range; LOS, length of stay; VSARR-RT, valve-sparing aortic root replacement—reimplantation technique.

In total nine different surgeons were included in this analysis. One surgeon performed 29 cases, the second most frequent surgeon performed 27 cases, and the third most frequent surgeon performed 11 cases. The most frequently performing surgeon was at our institution for 5 years; the second most frequently performing surgeon was at our institution for 2 years; and the third most frequently performing surgeon for 7 years. The three most frequent surgeons performed the majority of the cases (67 cases, or 79%). The vast majority of procedures incorporated the use of a Valsalva graft (79 cases, or 93%). The remaining six procedures were performed by a single surgeon and used a straight Hemashield graft.

There were no operative mortalities, early valve failures, or postoperative strokes ( Table 2 ). One patient required a reoperation for bleeding. The median hospital stay was 6 (IQR 5–7) days.

### Late Outcomes

The median postoperative observation time was 6.4 years (IQR 3.1–7.6), for a total of 491 patient-years of follow-up. Kaplan-Meier analysis demonstrated survival of 100% at 2 years and 99% (95% confidence interval [CI]: 97–100%) at 8 years postoperatively. Three patients died over the entire follow-up; two patients died from lung adenocarcinoma and one patient died from small cell carcinoma.

**Fig. 1:**
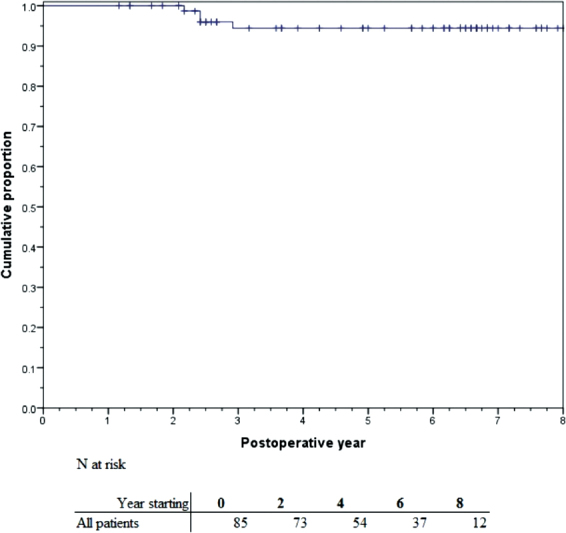
Kaplan-Meier estimation of reoperation-free survival.

Fig. 1 shows the Kaplan-Meier estimation for reoperation-free survival; overall there were three reoperations during our study observation, all of which occurred between years 2 and 3 postoperatively. Reoperation free-survival was 94.5% (95% CI: 89.2–99.7%) at 8 years.

Follow-up echocardiographic data were available for 80/85 patients ( Table 3 ). Freedom from endocarditis, pseudoaneurysm, or aneurysm development was 100% at 8 years. Moderate or greater AI developed during the postoperative period in a total of 16 patients and was central in 90% ( *n* = 14) of the patients, and eccentric in the remaining 10% of the patients.

**Table 3:** Echocardiographic follow-up

Variables	Patients ( *n* = 85), No. (%)
Follow-up echo available	80 (94.1)
Grade of AI	
None	18 (22.5)
Trace/trivial	28 (35.0)
Mild	18 (22.5)
Moderate	12 (15.0)
Severe	4 (5.0)

Abbreviations: AI, aortic insufficiency.

At last echocardiographic follow-up, 95% of patients were free of > 3+ AR and 82% were free of > 2+ AR. Of the four patients who had > 3+ AR, three underwent reoperations and received prosthetic valves and one is being clinically monitored. Twelve patients with moderate AR are being serially monitored by transthoracic echocardiogram at 6-month intervals.

## Discussion

Aortic root aneurysms require prophylactic repair, but careful consideration of repair type is essential. Valve-sparing root replacement is ideally suited for young patients with aortic root aneurysms, as long as the surgeon can offer a high degree of freedom from repair failure. The alternative option of composite valve graft replacement mandates patients to long-term therapeutic anticoagulation when using mechanical valves, durability concerns for bioprosthetic valves, and risk of thromboembolic complications with both valve types. 5 6 However, valve graft conduits as described in the original Bentall-DeBono procedure have demonstrated excellent long-term survival and a low rate of aortic reoperation. 1

What conclusions can we come to regarding the safety and outcomes of the VSARR-RT? Based on our results and of others, it is clear that this is a safe operation. 3 6 7 8 9 10 In this study we have demonstrated strong early- and midterm results for VSARR. We had no early mortality and no valve-related mortality over a total of 491 patient years, and our freedom from reoperation was 95.8%. In comparison, studies investigating the outcomes of patients undergoing mechanical valve composite grafts demonstrate long-term event-free survival to be approximately 40%. 11 12 13 Our midterm outcomes are in line with prior studies, thereby supporting the mortality benefit of valve-sparing technique over composite valve graft surgery.

We had a relatively heterogeneous population, including 46% with connective tissue disorders. There were a total of 4 patients out of 85 who developed greater than 3+ AI, 16 patients with moderate or greater AI, and 3 who required reoperation for repair failure. Of these three patients, only one had a connective tissue disorder, suggesting that our results are comparable in both a population with a connective tissue disorder and one without. Additionally, none of the patients with a bicuspid aortic valve required a repair, nor did any of the four patients who undergo emergency repair for acute aortic dissection. We were not able to identify a specific factor that contributed to our reoperation rate, but it is reassuring that connective tissue disorder, bicuspid valve, or emergency status did not lead to higher rates of reoperation. Uniquely, other studies have not borne out this finding regarding emergency cases. 14 15 This may be explained by low sample size ( *n* = 4) or surgical technique. Additionally, myxomatous changes may remain in the aortic cusps of patients with connective tissue disorders, causing concern for late failures with valve-sparing root replacement techniques. 16 This is not borne out by our results here, but longer-term follow-up is critical to definitively address this concern.

Despite being a single-center study, it has some advantages. It shows that the procedure can be performed safely and with sound results by different surgeons. Our study included surgeries performed by nine different surgeons. Three different surgeons performed the majority of cases, and six other surgeons also successfully performed the operation. This is in comparison to most of the existing literature. Many reports describe the experience of a single surgeon, thereby limiting the applicability of the results to a broader audience. 17 18 Some cardiac surgeons may not attempt to reproduce difficult technical procedures based on single-surgeon case series. Institutional knowledge is difficult to quantify, but our experience suggests that co-surgeon availability for intraoperative decision-making may contribute to reproducibility and acceptable results. It also reflects how surgeons with greater experience can help contribute toward good reproducibility of results. 19

This single-center retrospective review has several limitations. Our patients were predominantly young males with elective surgical status. Results observed here may not be generalizable to other populations. One patient was lost to survival follow-up, and five were lost to echocardiographic follow-up. It is possible that in such a small series, these missing cases were not missing at random, and we may be underestimating events. Conversely, there were more follow-up observations for those patients with known connective tissue disorders, which may cause overestimation of postoperative events compared with patients without these risk factors. Our findings should be interpreted with these cautions in mind.

In summary, prophylactic root and valve preservation using the David reimplantation is safe and has excellent early- and midterm outcomes. Longer-term follow-up will be critical in identifying the success rate of these procedures.
